# Examining a structural equation model of peace of mind, gratitude, and positive reappraisal in predicting psychological well-being among college students

**DOI:** 10.1186/s40359-025-03445-x

**Published:** 2025-09-29

**Authors:** Wei Du, Limin Liu

**Affiliations:** Psychological Health Education and Counseling Center, Jiaozuo Normal College, Jiaozuo, 454000 China

**Keywords:** Gratitude, Positive reappraisal, Peace of mind, Psychological well-being, College students, Mental health, Structural equation modeling

## Abstract

This study examined the relationships among gratitude, positive reappraisal, peace of mind, and psychological well-being, with a focus on their roles in being associated with mental health among Chinese college students. A cross-sectional design was employed, with 336 undergraduate students from various disciplines recruited through convenience sampling from four universities in mainland China. Participants completed validated self-reported measures of gratitude (GQ-6), positive reappraisal (CERQ), peace of mind (PoMS), psychological well-being (SPWB), and perceived academic pressure. Confirmatory factor analyses supported the construct validity of the scales, and structural equation modeling (SEM) revealed significant direct effects of gratitude, positive reappraisal, and peace of mind on psychological well-being. Additionally, peace of mind was found to mediate the relationships between gratitude, positive reappraisal, and well-being, suggesting that emotional stability and inner tranquility are important pathways to enhanced mental health. Clinically, these findings underscore the potential for integrating gratitude and positive reappraisal into therapeutic interventions to cultivate peace of mind and support mental health outcomes. Such interventions could be particularly valuable for students navigating high-pressure academic environments.

**Supplementary Information:**

The online version contains supplementary material available at 10.1186/s40359-025-03445-x.

## Introduction

Positive psychology provides valuable frameworks for understanding factors associated with human flourishing, particularly psychological well-being (PWB) [3]. For college students navigating a complex environment with numerous stressors [1], identifying personal resources that support PWB is crucial. Among these resources, dispositional traits like gratitude [4] and cognitive strategies such as positive reappraisal [5] are increasingly recognized for their association with mental health. Grounded in an emotion regulation framework, this study explores how these constructs interrelate. Understanding the nature of these relationships is essential for developing a more nuanced picture of a flourishing student experience.

We chose an emotion regulation perspective as a guiding framework because it can parsimoniously connect our variables of interest. Gratitude, the appreciation of the good in one’s life [6], can be viewed as a regulatory practice of attentional deployment, where focus is intentionally shifted toward positive experiences. Positive reappraisal, the reinterpretation of stressful events more favorably [5], is a well-established cognitive change strategy that mitigates negative affect [9]. Empirical studies consistently report positive associations between gratitude and well-being outcomes [7, 8], and between positive reappraisal and effective coping [9]. However, much of this research establishes direct links [1, 7, 9, 10], leaving a gap in understanding the intermediary variables that might help explain these associations. Specifically, the potential for a shared underlying process or affective state that connects these distinct regulatory strategies to PWB remains underexplored.

The present study aims to clarify these relationships by examining a model in which peace of mind is positioned as a potential statistical mediator. Peace of mind, a state of inner tranquility and emotional stability [11], is linked with positive psychological outcomes [12, 13]. We propose that the associations of gratitude and positive reappraisal with PWB might be partially explained by their shared positive relationship with peace of mind. Our objective is not to establish causality, but rather to investigate a plausible explanatory model based on cross-sectional data. Specifically, we pose the following research questions: (1) Are gratitude and positive reappraisal independently associated with PWB in our sample of college students? (2) To what extent is the relationship between gratitude and PWB statistically mediated by peace of mind? (3) To what extent is the relationship between positive reappraisal and PWB statistically mediated by peace of mind?

This research offers several contributions. First, it investigates the understudied concept of peace of mind within the context of student well-being in China. Second, it moves beyond direct associations by examining a mediational model. By exploring these pathways, this study provides a more detailed understanding of how these positive psychology constructs are interrelated. Finally, by elucidating the nature of the association between these regulatory strategies and inner calm, our findings have practical implications for wellness initiatives. The results may help inform interventions that target gratitude and positive reappraisal practices, with the understanding that an associated outcome of such practices may be an enhanced sense of peace of mind and, in turn, greater overall well-being.

### Dimensions and determinants of student well-being

Psychological well-being, a broad term encompassing mental health and flourishing, is central to student success [3, 81]. It extends beyond merely the absence of distress, embracing a range of subjective experiences, emotional regulation, purpose, and overall quality of life [1, 82]. Ryff’s model [3] identifies six key dimensions of psychological well-being: self-acceptance, positive relationships, autonomy, environmental mastery, purpose in life, and personal growth. Individuals who exhibit high psychological well-being typically experience greater life satisfaction, positive emotions, and a stronger sense of meaning in their lives [14]. Moreover, research has linked high levels of well-being to better physical health, quicker recovery from illness, and increased longevity [15].

Numerous factors influence student well-being, including individual characteristics, social connections, and environmental influences [16]. Personal attributes such as self-esteem, optimism, and resilience significantly contribute to well-being [1]. Additionally, the presence of supportive social relationships, a sense of belonging, and a positive social environment are crucial [17]. Interventions such as mindfulness meditation, positive psychology practices, and cognitive-behavioral therapy have been shown to enhance well-being and coping skills effectively [18, 19]. Delving deeper, research reveals a complex interplay of psychological factors that influence well-being. Studies by Di Fabio and Bucci [20] and Morales-Rodríguez et al. [21] demonstrate strong positive correlations between life satisfaction, self-esteem, optimism, and psychological well-being among students. These findings highlight the interconnectedness of various affective states and overall well-being. Furthermore, self-efficacy and emotional intelligence (EI) consistently emerge as significant contributors to well-being [22, 23]. According to Costa et al. [24], both of these factors positively impact college students’ well-being. Students who possess strong emotional awareness, self-management skills, and a belief in their abilities are more likely to experience greater well-being, underscoring the potential benefits of interventions aimed at cultivating these skills.

The importance of positive relationships cannot be overstated. Murray-Harvey [25] emphasizes the influence of supportive school relationships on student outcomes. Meanwhile, Brunsting et al. [26] delve deeper, exploring specific sources of perceived social support for international students. They find a significant connection between positive social-emotional experiences and well-being, suggesting that fostering a sense of belonging and social connection is crucial for student mental health.

### The role of gratitude in enhancing mental health

Gratitude, a positive emotion experienced when individuals acknowledge and appreciate the good in their lives, has garnered significant attention in psychological research due to its potential impact on well-being and mental health [4, 83]. Encompassing feelings of thankfulness, appreciation, and recognition of the positive aspects of one’s life, gratitude is acknowledged regardless of the magnitude of these positive aspects [6]. Numerous studies have demonstrated the beneficial effects of gratitude on various facets of psychological well-being. Individuals who regularly engage in gratitude practices report higher levels of life satisfaction, happiness, and positive affect [7, 27, 84]. Moreover, gratitude is associated with lower levels of depression, anxiety, and stress, serving as a protective factor against mental health problems [6, 8].

One mechanism through which gratitude enhances well-being is by promoting positive social interactions and strengthening relationships. Grateful individuals tend to be more empathetic, compassionate, and prosocial, fostering a sense of connection and belongingness with others [28, 29]. Expressing gratitude towards others can strengthen interpersonal bonds and deepen relationships, leading to greater social support and satisfaction [30]. Additionally, gratitude interventions, such as keeping gratitude journals or writing gratitude letters, have been found to be effective in promoting well-being and mental health [77]. These interventions encourage individuals to focus on the positive aspects of their lives and cultivate a mindset of appreciation [4]. Research suggests that even brief and simple gratitude exercises can have lasting effects on psychological functioning and subjective well-being [31].

In addition to these interpersonal benefits, neuroscientific evidence highlights gratitude’s impact on brain regions associated with reward processing, empathy, and emotional regulation [32, 33]. This neurobiological foundation underscores gratitude’s role in fostering emotional balance and social connectedness, key contributors to psychological well-being [34]. The positive association between gratitude and well-being often surpasses other predictors, such as hope and optimism, reinforcing its unique influence on mental health outcomes [35, 36]. Furthermore, gratitude amplifies the positive effects of social support, acting as a bridge that strengthens relationships and enhances well-being [37]. These benefits are consistent across diverse student populations, including high school and university students [10, 35].

Beyond its direct effects, gratitude’s relationship with well-being can be understood through its role in fostering peace of mind—a state of emotional tranquility and inner calm [11]. Gratitude encourages individuals to focus on positive aspects of their lives while diminishing the impact of negative emotions, creating an emotional balance conducive to peace of mind. Fredrickson’s broaden-and-build theory provides a useful framework for understanding this process. According to the theory, positive emotions such as gratitude expand individuals’ cognitive and emotional capacities, enabling them to build resilience and attain a stable sense of inner peace [12].

Peace of mind serves as a psychological buffer against stress by enhancing emotional regulation and equipping individuals to face challenges with greater resilience [13]. It mediates the relationship between gratitude and psychological well-being by converting gratitude’s immediate emotional benefits into sustained mental health improvements. For example, students who practice gratitude often experience reduced rumination and better stress management, which contribute to improved overall well-being. Evidence supports this mediation mechanism, suggesting that peace of mind acts as the connecting pathway that links gratitude to long-term psychological health benefits [13].

The mediating role of peace of mind in the relationship between gratitude and well-being aligns with findings from diverse settings. For example, Jun et al. [38] demonstrated that gratitude could buffer depressive symptoms in high-stress environments, such as nursing. These findings underscore the versatility of gratitude as a psychological tool with applications across populations. By fostering emotional stability and inner calm, gratitude interventions tailored to the unique challenges of students have the potential to enhance mental health outcomes effectively.

### Peace of mind and its influence on well-being

Peace of mind, a state often sought after by individuals, is a complex and multifaceted concept that has garnered attention across various fields of study [11, 78]. In the realm of psychology, it is often associated with feelings of contentment, tranquility, and emotional well-being [11]. Moreover, peace of mind has been linked to positive outcomes such as improved physical health, enhanced resilience, and better coping mechanisms in the face of stressors [12, 13]. One key factor contributing to peace of mind is the ability to manage and regulate one’s emotions effectively. Research by Gross [39] suggests that individuals who possess strong emotional regulation skills are better equipped to navigate challenging situations and maintain a sense of inner calmness. Similarly, mindfulness practices have been shown to promote peace of mind by fostering present-moment awareness and acceptance of one’s experiences [40].

Furthermore, social support plays a crucial role in fostering peace of mind. Studies have demonstrated that having a strong network of supportive relationships can buffer against the negative impact of stressors and promote psychological well-being [41, 42]. This highlights the importance of interpersonal connections in cultivating a sense of security and peace within individuals. In addition to psychological factors, environmental influences also contribute to peace of mind. For instance, access to safe and stable living conditions, such as secure housing and neighborhoods, has been associated with higher levels of subjective well-being [2]. Similarly, environmental factors such as natural landscapes and green spaces have been shown to promote relaxation and reduce stress levels [43].

The concept of “peace of mind” emerges as a significant factor influencing student well-being, though research explores it from various angles. Studies by Sophie et al. [44] and Yu et al. [45] link peace of mind directly to subjective well-being and positive emotions, suggesting that a state of inner tranquility fosters a more positive outlook. Further research delves into the potential consequences of peace of mind for student behavior. Datu’s work [46, 47] in the Philippine context demonstrates a positive association between peace of mind and academic engagement and achievement. Students experiencing peace of mind appear to be more motivated and successful academically. Therefore, while not a core variable in our model, the established link between peace of mind and academic achievement highlights its practical relevance for a student population facing high performance expectations. Interestingly, Constantinou et al. [48] highlight the potential for interventions promoting “peace of mind” (PEACE) to enhance psychological support for medical students. This suggests that fostering a sense of inner calm may be particularly crucial for student populations facing high levels of stress.

It is important to note that the research on peace of mind is not limited to student populations. Fu et al. [49] explore the connection between peace of mind and life satisfaction among dentists, suggesting a broader applicability of this concept to mental well-being. In conclusion, the concept of peace of mind holds promise for understanding and promoting student well-being. Future research could explore the specific mechanisms by which peace of mind fosters positive emotions, academic engagement, and overall well-being. Additionally, investigations into interventions designed to cultivate peace of mind among students could provide valuable tools for supporting mental health in educational settings.

### Positive reappraisal as a coping mechanism

Positive reappraisal, a cognitive coping strategy that involves reinterpreting stressful or negative events in a more positive light, has been extensively studied within the context of resilience and psychological well-being [5, 85]. This adaptive mechanism allows individuals to shift their perspective, finding meaning, growth, or opportunities for personal development amid challenging circumstances [9]. Research consistently indicates that those who engage in positive reappraisal experience fewer negative emotions and greater psychological resilience when facing adversity [9, 54, 79]. By reframing stressful situations in a more positive or meaningful way, individuals can significantly mitigate the impact of stressors on their mental health and well-being [5]. Moreover, positive reappraisal has been associated with improved coping strategies, adaptive functioning, and overall life satisfaction [50].

A key aspect of positive reappraisal is its capacity to help individuals find silver linings or perceive potential benefits in difficult circumstances [80]. This cognitive restructuring process allows individuals to focus on their personal strengths, resources, and opportunities for growth, fostering a sense of empowerment and control [51]. Additionally, positive reappraisal facilitates a sense of coherence and meaning-making, enabling individuals to construct narratives that emphasize resilience, learning, and personal development in the face of adversity [52]. Positive reappraisal is not only beneficial psychologically but also physiologically. Studies have demonstrated that individuals who engage in positive reappraisal exhibit lower levels of physiological arousal and stress reactivity, along with enhanced immune functioning [9, 53]. Furthermore, this coping strategy has been linked to greater psychological well-being, including higher levels of self-esteem, optimism, and positive affect [50].

However, the effectiveness of positive reappraisal can vary depending on individual differences, contextual factors, and the nature of the stressor. While some individuals may naturally gravitate toward positive reappraisal as a coping strategy, others may require training or intervention to develop this skill [9]. Additionally, certain stressors may be more amenable to positive reappraisal than others, highlighting the need for flexibility and adaptability in coping strategies [51].

Several studies underscore the potential of positive reappraisal, particularly as a cognitive emotion regulation strategy, to enhance student well-being [54–56]. Studies by Riepenhausen et al. [54] and Shum et al. [57] provide robust evidence for this association across diverse age groups, including early adolescence and university students. Positive reappraisal appears to function in two key ways. First, it acts as a buffer against negative emotions, particularly those stemming from academic challenges, suggesting that students who can reframe stressful situations in a more positive light experience less psychological distress [55]. Second, research by Pogrebtsova et al. [56] demonstrates that interventions promoting positive reappraisal can lead to daily improvements in well-being among university students.

Interestingly, Haga et al. [58] highlight the potential for cultural variations in the effectiveness of positive reappraisal. Future research could explore how cultural contexts influence students’ use and benefits of this strategy. In conclusion, a growing body of research suggests that positive reappraisal offers a promising avenue for promoting student well-being. Further investigation into the mechanisms of this association and the development of culturally-sensitive interventions could optimize the use of positive reappraisal to support student mental health.

### The current study

This study aims to explore the intricate relationships among gratitude, positive reappraisal, peace of mind, and psychological well-being in a student population. Building on extensive research in positive psychology, the study proposes a theoretical model that integrates direct and indirect pathways, providing a nuanced understanding of how these constructs interrelate.

Gratitude is widely recognized as a significant predictor of psychological well-being, fostering positive emotions, life satisfaction, and happiness while serving as a buffer against mental health challenges such as depression, anxiety, and stress [1, 7, 27]. Therefore, we hypothesize a direct positive association between gratitude and psychological well-being (**H1**).

Similarly, positive reappraisal, a cognitive emotion regulation strategy, has been shown to mitigate the adverse effects of stress by enabling individuals to reinterpret challenging situations in a more constructive light. This approach enhances psychological resilience, self-esteem, and optimism, leading to improved well-being [5, 9, 50]. Based on these findings, we hypothesize a direct positive association between positive reappraisal and psychological well-being (**H2**).

While these direct relationships are well-documented, this study introduces peace of mind as a potential mediator, offering a more comprehensive perspective. Peace of mind, defined as a state of emotional tranquility and inner contentment, has been linked to enhanced mental health outcomes, including reduced stress and greater life satisfaction [11, 13]. We posit that gratitude may cultivate peace of mind by encouraging appreciation for life’s positive aspects and strengthening social bonds [28, 29]. This peace of mind, in turn, may facilitate greater psychological well-being (**H3**).

**Fig. 1:**
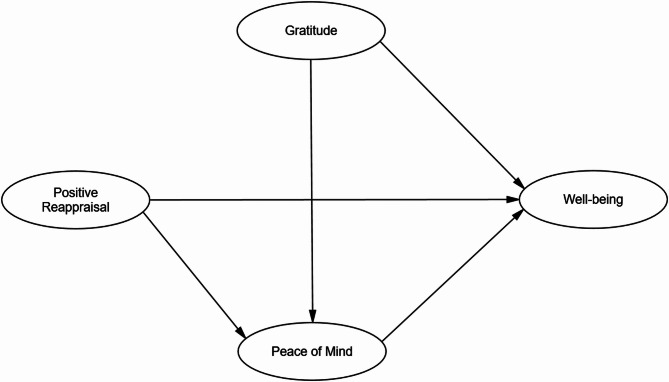
The conceptual model

Similarly, positive reappraisal is hypothesized to promote peace of mind by helping individuals find meaning, personal growth, and emotional balance in adverse situations [52]. This inner calmness could then mediate the relationship between positive reappraisal and psychological well-being (**H4**). The conceptual framework is visually represented in the model diagram (Fig. 1).

## Methods

### Participants and procedures

This study employed a cross-sectional design to investigate the relationships among gratitude, positive reappraisal, peace of mind, and psychological well-being among Chinese college students. Participants were undergraduate students recruited through a non-probability convenience sampling approach from four universities located in four provinces of mainland China: Beijing, Guangdong, Sichuan, and Shandong. These universities were selected to intentionally include participants from diverse geographic contexts, spanning the eastern, southern, western, and northern regions of the country. While this multi-region approach sought to increase the diversity of the sample, we acknowledge that the use of convenience sampling means the findings may not be generalizable to the broader Chinese student population.

The study included 336 undergraduate students (54.2% female; M age = 21.16, SD = 1.79, range = 18–22). Eligible participants were enrolled in an undergraduate program, provided informed consent, and reported no significant history of psychological or medical conditions. The final sample was academically and socio-economically diverse, representing a range of disciplines and both urban and rural backgrounds. Recruitment prioritized second- and third-year students to capture a cohort experiencing typical academic demands. Our target sample size was determined based on established recommendations for structural equation modeling (SEM), ensuring a statistical power of 0.90 to detect medium-to-large effects in our mediational model [73, 86]. The final sample of 326 participants exceeded the minimum required threshold for reliable estimation.

With the cooperation of university faculty, students were invited to participate during class sessions. Data were collected online between late October and mid-November 2023 using the Wjx platform, a secure survey tool. After providing informed consent, participants voluntarily completed the 10–15 min battery of questionnaires without receiving any incentives.

Rigorous quality control measures were implemented to ensure data reliability. Completion times were automatically logged via the Wjx platform. Responses completed in under five minutes, considered insufficient time for thoughtful engagement with the survey content, were reviewed and subsequently excluded (*n* = 6). Additionally, participants failing an embedded attention check item designed to monitor response quality were also excluded (*n* = 4). In total, 10 participants were removed based on these quality criteria. These procedures helped ensure the dataset’s reliability and validity for the subsequent SEM analyses.

### Instruments

All scales underwent a standard translation and back-translation process and were pilot-tested with 30 students to ensure cultural relevance and clarity. The construct validity of each measure was confirmed using Confirmatory Factor Analysis (CFA).

### Psychological well-being

We measured psychological well-being with the 18-item Scale of Psychological Well-being (SPWB) [14], an instrument validated for use in China [87]. The SPWB assesses six core dimensions of flourishing: autonomy, environmental mastery, personal growth, positive relations, purpose in life, and self-acceptance. In our sample, the scale showed strong internal consistency (α = 0.86), and CFA results confirmed its construct validity (χ2/*df* = 2.24, CFI = 0.93, TLI = 0.91, RMSEA = 0.061, SRMR = 0.049).

### Gratitude

Gratitude was assessed using an adapted version of the 6-item Gratitude Questionnaire (GQ-6) [59], which has established validity in Chinese samples [60]. For this study, the original 7-point response scale was adjusted to a 5-point Likert format to better align with local survey practices. The adapted scale demonstrated high reliability (α = 0.87) and good construct validity in our analysis (χ2/*df* = 2.31, CFI = 0.95, TLI = 0.93, RMSEA = 0.062, SRMR = 0.045).

### Positive reappraisal

Positive reappraisal was measured with the 4-item subscale from the Cognitive Emotion Regulation Questionnaire (CERQ) [61], whose Chinese version is well-validated [88]. This subscale assesses the tendency to reframe negative events in a positive light, with items such as, “I think I can become a stronger person as a result of what happened.” The measure exhibited good reliability in our study (α = 0.81), and CFA supported its construct validity (χ2/*df* = 1.96, CFI = 0.96, TLI = 0.94, RMSEA = 0.055, SRMR = 0.038).

### Peace of mind

To gauge inner peace, we used the 7-item Peace of Mind Scale (PoMS) [62], previously validated in the Chinese context [89]. The scale measures feelings of internal harmony and tranquility with items like, “I have peace and harmony in my mind.” The PoMS displayed excellent internal consistency (α = 0.89) and strong construct validity in our sample (χ2/*df* = 2.12, CFI = 0.94, TLI = 0.92, RMSEA = 0.059, SRMR = 0.042).

### Statistical analysis

A robust statistical framework was employed to meticulously analyze the data and rigorously assess the hypothesized relationships. Our analysis proceeded in a systematic, multi-step process to ensure the highest possible quality and reliability of the findings. To mitigate potential common method bias (CMB) inherent in self-reported data [63], a multifaceted approach was implemented. We first conducted preliminary data screening, followed by tests of our measurement model, and finally, evaluation of the full structural model.

The initial steps focused on data screening and quality control, which included assessing for missing data, identifying outliers, and confirming the robustness of our data preparation choices. This was a critical precursor to our main analyses. Next, to validate our scales and ensure they accurately measured the intended constructs, we performed convergent and discriminant validity assessments [64]. Confirmatory factor analysis (CFA) was then utilized to evaluate the measurement model’s adequacy [65]. Descriptive statistics and reliability coefficients were also computed to provide insights into central tendency, dispersion, and normality of the data distribution.

Structural equation modeling (SEM) was subsequently deployed to explore the hypothesized relationships within the structural model. Various fit indices were employed to assess the model’s overall tenability. These included the root mean square error of approximation (RMSEA), with values closer to 0.06 indicating an acceptable fit [66]. Additionally, the standardized root mean square residual (SRMR) below 0.08 was considered satisfactory [66]. We aimed for a chi-square to degrees-of-freedom ratio between 1 and 3, and a comparative fit index (CFI) exceeding 0.90 [67]. Furthermore, other criteria encompassed a goodness-of-fit index (GFI) exceeding 0.90 and a Tucker-Lewis index (TLI) surpassing 0.90 [68].

## Results

### Initial data quality and suitability checks

Preliminary data analysis confirmed the dataset’s suitability for structural equation modeling (SEM) [69]. Little’s test indicated that the small amount of missing data (2.5%) was missing completely at random (*p* = .08), so imputation was not required [70, 71]. We then screened for outliers using Mahalanobis distance and standardized residuals [72]. This process identified two influential outliers, which were removed to maintain the integrity of the analysis. Three moderately deviating cases were retained after winsorizing to minimize their impact. A robustness check confirmed that winsorizing did not substantially alter the primary model’s path coefficients, reinforcing the stability of our findings.

### Measurement model

We evaluated the four-factor measurement model using CFA in AMOS. While the chi-square test was significant (χ2 = 225.43, *p* < .001), a common result in larger samples [66, 73], a holistic assessment of the other fit indices confirmed the model’s validity. The key indices all met or exceeded recommended benchmarks for a good fit: CFI = 0.931, TLI = 0.918, RMSEA = 0.062, and SRMR = 0.047 [66]. These results support the proposed factor structure, indicating that the observed variables were reliable indicators of their respective latent constructs (gratitude, positive reappraisal, peace of mind, and psychological well-being).

Consequently, given our reliance on self-report data collected at a single time point, we conducted several procedural and statistical checks to assess for common method bias (CMB) [74]. Procedurally, we used well-validated scales with distinct formats. Statistically, we employed a series of post-hoc tests to confirm that CMB was not a significant concern.

First, Harman’s single-factor test [75] showed that the first factor accounted for only 39.06% of the variance, well below the 50% threshold. Second, a theoretically unrelated marker variable (“preference for outdoor activities”) showed negligible correlations with our main study variables (*r*s = 0.02 to 0.05) [76]. Third, the Fornell-Larcker criterion [64] supported the discriminant validity of our constructs. Finally, a confirmatory factor analysis revealed that a single-factor model fit the data poorly (e.g., RMSEA = 0.122, CFI = 0.674), while our proposed four-factor model demonstrated a good fit. Collectively, these comprehensive checks provide strong evidence that our results were not substantially influenced by common method bias.

### Descriptive statistics and reliability

Table 1 presents the descriptive statistics for the study variables. All measures demonstrated good to excellent internal consistency, with Cronbach’s alpha coefficients ranging from 0.81 to 0.89. An examination of skewness and kurtosis statistics revealed no significant deviations from a normal distribution, confirming that the data met the assumptions for the subsequent analyses [72].

**Table 1:** Descriptive statistics and internal consistency

Construct	M (SD)	Cronbach’s α	Skewedness	Kurtosis
Gratitude	3.82 (0.74)	0.87	-0.32	0.21
Positive Reappraisal	4.15 (0.68)	0.81	-0.18	0.09
Peace of Mind	4.38 (0.59)	0.89	-0.07	-0.12
Well-being	3.97 (0.71)	0.86	-0.25	0.14

Table 2 shows the correlations and validity metrics for the latent constructs. All inter-construct correlations were positive and statistically significant (*p* < .01). Convergent validity was supported, as the Average Variance Extracted (AVE) for each construct was well above the recommended 0.50 threshold, indicating that the measures captured a substantial amount of variance [64, 69]. Discriminant validity was also established, as the square root of each construct’s AVE (the bolded diagonal values in Table 2) was greater than its correlation with any other variable, confirming the distinctiveness of the constructs.

**Table 2:** Convergent and discriminant validity and correlations

Construct	AVE	1	2	3	4
1. Gratitude	0.72	0.85			
2. Positive Reappraisal	0.68	0.52**	0.83		
3. Peace of Mind	0.81	0.48**	0.45**	0.90	
4. Well-being	0.74	0.56**	0.42**	0.51**	0.86

Note. Square root values of AVE are shown on the diagonal (bold). Correlation values are below the diagonal and statistically significant at *p* < .01 level (**)

### Structural equation modeling

We used structural equation modeling (SEM) in AMOS (v. 26.0) to test our hypothesized mediational model. The model demonstrated an acceptable fit to the data: χ2(120) = 321.47, *p* < .001; χ2/*df* = 2.68; CFI = 0.917; TLI = 0.892; RMSEA = 0.071; SRMR = 0.054. Although the chi-square test was significant, a common finding in larger samples, the other fit indices collectively support the model’s adequacy [66].

**Table 3:** Path coefficients of SEM results

Path	Direct Effects	Indirect Effects	Total Effects
Gratitude → Well-being	*β* = 0.334	*β* = 0.167	*β* = 0.501
	BC-CI [0.250, 0.418]	BC-CI [0.120, 0.210]	BC-CI [0.420, 0.580]
	*p* < .001	*p* < .001	*p* < .001
Positive Reappraisal → Well-being	*β* = 0.274	*β* = 0.140	*β* = 0.414
	BC-CI [0.200, 0.348]	BC-CI [0.100, 0.180]	BC-CI [0.330, 0.490]
	*p* < .001	*p* < .001	*p* < .001
Peace of Mind → Well-being	*β* = 0.446		
	BC-CI [0.370, 0.522]		
	*p* < .001		
Gratitude → Peace of Mind	*β* = 0.375		
	BC-CI [0.300, 0.450]		
	*p* < .001		
Positive Reappraisal → Peace of Mind	*β* = 0.314		
	BC-CI [0.240, 0.388]		
	*p* < .001		

**Fig. 2:**
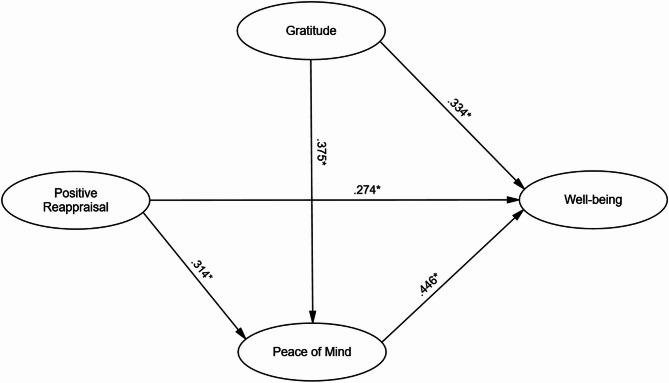
The model of peace of mind, gratitude, and positive reappraisal in predicting psychological well-being. All path coefficients are significant at *p* < .001

The standardized path coefficients, detailed in Table 3; Fig. 2, supported all primary hypotheses. We found significant direct positive associations of gratitude (β = 0.334, *p* < .001) and positive reappraisal (β = 0.274, *p* < .001) with psychological well-being. Crucially, the analysis revealed significant indirect paths to well-being through peace of mind from both gratitude (β = 0.167, *p* < .001) and positive reappraisal (β = 0.140, *p* < .001). These results confirm the significant mediating role of peace of mind in the relationships between these positive traits and well-being.

To further validate our proposed structure, we compared it against two alternative models. A “direct effects only” model that omitted peace of mind as a mediator showed a substantially poorer fit (e.g., RMSEA = 0.091; CFI = 0.882), as did a null model. The superior fit of our hypothesized model reinforces its plausibility and highlights the importance of peace of mind in the overall structure.

Finally, we explored two additional model configurations to better understand the interplay between the variables. One model testing positive reappraisal as a mediator of the gratitude-to-well-being relationship also fit the data well and revealed a significant indirect effect (β = 0.125, *p* < .001). Another model showed that peace of mind’s effect on well-being was partially mediated by gratitude (β = 0.141, *p* < .001) and positive reappraisal (β = 0.098, *p* < .001). While these alternative models had slightly weaker fit indices, their acceptable fit suggests a complex and potentially reciprocal set of relationships among these constructs that warrants further investigation.

### Measurement invariance testing

To ensure our model operated equivalently for male and female students, we conducted a multi-group measurement invariance test. The analysis proceeded sequentially, testing for configural, metric, scalar, and strict invariance. The baseline configural model demonstrated a good fit to the data (χ2(240) = 530.45, *p* < .001, CFI = 0.912, RMSEA = 0.058), indicating that the factor structure was consistent across genders.

Subsequently, more constrained models testing for metric (equal factor loadings), scalar (equal intercepts), and strict (equal residual variances) invariance also showed a good fit. Crucially, the change in the Comparative Fit Index (ΔCFI) between each successive model was negligible (ΔCFI ≤ 0.002), providing strong support for full measurement invariance. These results confirm that the constructs were understood and measured equivalently across male and female students, supporting the generalizability of our findings.

## Discussion

This study examined the relationships among gratitude, positive reappraisal, peace of mind, and psychological well-being among Chinese college students. Four hypotheses were tested, and the findings provided robust support for a model in which peace of mind statistically mediates the relationships of gratitude and positive reappraisal with psychological well-being. These findings advance our understanding of the interrelationships that are associated with mental well-being among students in a Chinese context.

### Gratitude and psychological well-being

Regarding Hypothesis 1 (H1), the results demonstrated a significant direct positive association between gratitude and psychological well-being. This finding is consistent with a well-established body of literature showing that gratitude is associated with positive emotions, life satisfaction, and happiness while being linked to buffering against mental health challenges such as depression, anxiety, and stress [1, 7, 27]. From the perspective of Fredrickson’s broaden-and-build theory [12], gratitude encourages individuals to focus on the positive aspects of life, which may reduce the prominence of negative thought patterns and emotional distress [4, 6]. This optimistic outlook is associated with enhancing students’ resilience and their ability to cope effectively with study-related stressors [7, 28].

In educational contexts, gratitude also co-occurs with greater emotional support and social integration, which are key to mental health. In line with the “build” component of Fredrickson’s theory, grateful individuals are more likely to express appreciation and empathy, which is related to stronger interpersonal bonds and a sense of belonging [30, 36]. For Chinese students, gratitude aligns with collectivist cultural norms that emphasize relational interdependence and social harmony, which may further explain its positive link with well-being [37]. Moreover, gratitude is connected to the benefits of social support, reflecting a pattern of positive interactions and emotional resilience that is associated with enhanced psychological well-being and greater life satisfaction [36, 41]. Beyond these interpersonal effects, recent meta-analytic evidence suggests that gratitude interventions are an effective self-help strategy for reducing symptoms of depression and anxiety, further supporting our finding that gratitude is a strong correlate of psychological well-being [83, 84]. This indicates that the dispositional trait we measured is likely an important resource related to students’ mental health, especially in contexts of high academic stress.

### Positive reappraisal and psychological well-being

For Hypothesis 2 (H2), the study found that positive reappraisal is positively associated with psychological well-being. This finding aligns with prior research on cognitive emotion regulation strategies [5, 9, 50]. Interpreted through Gross’s process model of emotion regulation [39], positive reappraisal is a process through which students can reinterpret challenging situations, such as academic demands, in a constructive manner, reframing stressors as manageable and is associated with emotional stability and optimism [51, 54]. By shifting focus toward potential benefits or growth opportunities, positive reappraisal is linked to a reduction in negative emotional responses, such as frustration or anxiety, while being related to a more balanced emotional state [9, 52].

This study further demonstrated that peace of mind mediates the relationship between positive reappraisal and psychological well-being. Positive reappraisal appears to contribute to peace of mind by providing a framework for approaching challenges with greater emotional balance and clarity. Through the cognitive reframing of difficulties, individuals can report a greater sense of inner calm and resilience, which often co-exists with well-being [12, 13, 55]. In being associated with peace of mind, positive reappraisal is also associated with students maintaining psychological stability and better navigating academic stressors, which may explain its indirect association with enhanced well-being. This pattern is consistent with recent reviews highlighting that positive reappraisal is a key component of psychological resilience and well-being across diverse populations [54, 79, 80]. The ability to find a “silver lining” in a negative event, as described by Troy et al. [9], is not just a temporary emotional fix; it is a fundamental cognitive skill that, when consistently applied, is related to the mental resources connected with sustained psychological health and inner peace.

### Peace of mind as a mediator

Concerning Hypothesis 3 (H3), peace of mind was found to statistically mediate the relationship between gratitude and psychological well-being. The results of this study underscore peace of mind as a pivotal mediator linking gratitude and psychological well-being, warranting a deeper theoretical exploration of this association. Gratitude may be linked to peace of mind by encouraging students to focus on life’s positive aspects, which is associated with reducing the intensity of negative emotions such as frustration or despair, and is related to a sense of contentment and inner harmony [11, 13, 28, 29]. This shift in focus aligns explicitly with Fredrickson’s broaden-and-build theory [12], which posits that positive emotions broaden individuals’ cognitive and emotional resources, a process that may lead to resilience and a more balanced emotional state. Furthermore, gratitude is associated with stronger social bonds, which are a source of emotional support and are linked to reduced feelings of isolation. These strengthened interpersonal relationships are associated with enhanced feelings of security and belonging, both of which are related to peace of mind [30, 36]. This aligns with research suggesting that social connections are essential for the experience of peace of mind, particularly in contexts where interpersonal relationships play a central role in emotional stability [30, 42]. This dynamic is particularly pronounced in collectivist cultures, such as China, where harmonious relationships are a cornerstone of emotional well-being [37].

Gratitude also appears to be related to alleviating stress by being associated with adaptive emotional regulation, further connecting it to a tranquil state of mind [6, 11]. Grateful individuals are less likely to ruminate on negative experiences, which allows them to maintain emotional equilibrium and a sense of inner calmness [4, 6]. This finding is consistent with previous research showing that gratitude can buffer depressive symptoms in high-stress environments [38] by being associated with an internal state of peace that is resistant to external pressures. This mediating pathway highlights the association of gratitude with improved psychological well-being, a relationship that appears to be statistically explained, in part, by peace of mind, particularly in the high-stress environments typical of academic settings [13, 45].

Similarly, Hypothesis 4 (H4) was supported, with peace of mind statistically mediating the relationship between positive reappraisal and psychological well-being. Positive reappraisal is associated with peace of mind by being linked to individuals finding meaning and personal growth in adverse situations [52]. Through this cognitive strategy, students can report achieving a sense of emotional balance, reframing their stressors as manageable challenges rather than insurmountable obstacles [12, 13]. This process aligns directly with Gross’s theory of emotion regulation [39], which highlights the role of cognitive reappraisal in being associated with reduced emotional intensity and a greater sense of control over adverse situations [39]. By being linked to individuals finding meaning and growth in challenging circumstances, positive reappraisal is associated with emotional stability and a sense of coherence, both of which are integral to peace of mind [52]. Moreover, the ability to frame difficulties as opportunities for personal development is linked to psychological resilience, allowing individuals to maintain a state of inner tranquility even in high-stress contexts such as academic environments [50, 54].

Peace of mind may serve as a psychological buffer, and is associated with a reduced cognitive and emotional burden of stress and the ability for individuals to maintain a state of tranquility and focus [12, 44]. This buffering association is particularly relevant in educational settings, where students often face high levels of academic pressure. By being linked to this inner calmness, positive reappraisal not only appears related to immediate emotional well-being but also is associated with a stable foundation for long-term psychological health [44, 46]. This mediation pathway suggests that the associations of positive reappraisal extend beyond immediate emotional regulation, being linked to broader psychological well-being through its relationship with peace of mind [13, 58]. The findings on this mediating role are further supported by studies demonstrating that peace of mind is a significant predictor of positive emotions, subjective well-being, and even academic engagement, all of which are components of a flourishing life [44, 45, 47]. This provides a compelling, evidence-based reason for its central role in our model.

### Theoretical implications

From a theoretical standpoint, peace of mind appears to function as an important construct linking emotional regulation and psychological stability. It resonates with dimensions of eudaimonic well-being, such as autonomy, environmental mastery, and purpose in life ( [3, 14]), and our model suggests a potential pathway through which resources like gratitude and positive reappraisal are associated with fostering enhanced mental health ( [11, 58]). By being linked to emotional balance and psychological resilience ( [44, 46]), peace of mind may represent an affective state that is connected to the benefits of positive psychological traits and adaptive coping strategies.

This study contributes to positive psychology theory in three key ways. First, confirming peace of mind as a statistical mediator illuminates a specific psychological pathway that helps to explain the observed associations between gratitude, positive reappraisal, and college student well-being. This offers a more nuanced understanding compared to models focusing solely on direct effects. Second, our findings underscore the theoretical significance of peace of mind itself. It emerges as a distinct construct potentially bridging concepts across positive psychology, emotion regulation, and eudaimonic well-being frameworks [3, 11, 14, 39, 58], suggesting it warrants further conceptual attention. Third, the results support the relevance of existing theories, such as the broaden-and-build theory [12] and cognitive emotion regulation models [39], by illustrating how these frameworks can be used to understand the associations that lead toward inner calm and overall flourishing.

### Practical implications

The significant positive relationships observed in this study may point to several promising avenues for student wellness, though any practical applications must be considered speculative pending further research. First, the strong associations suggest that interventions aimed at cultivating gratitude (e.g., gratitude journaling) [4, 77] and enhancing positive reappraisal skills [9, 56] could be a valuable area for future experimental investigation.

Second, given the strong mediating role of peace of mind in our model, future intervention studies might also consider explicitly targeting the cultivation of inner tranquility. Practices derived from mindfulness traditions [40] or techniques aimed at reducing rumination could be valuable components to test. However, it is critical to emphasize that our cross-sectional design does not allow for causal inference. The efficacy of any such programs must be established through rigorous longitudinal or experimental research. Should such research yield positive results, educational institutions and counseling centers could then consider developing integrated programs based on that evidence.

### Limitations and future research

Although this study provides valuable insights, several limitations should be acknowledged. First, the cross-sectional design captures data at a single point in time, limiting our ability to infer causal relationships. While our model is theoretically grounded, longitudinal or experimental designs are needed to establish causality. Second, our reliance on a convenience sampling strategy is a key limitation. Because participants were not randomly selected, the sample may not be representative of the entire Chinese college student population, and the findings should be interpreted with caution regarding their generalizability. Future research should aim to replicate these findings using probability sampling methods to obtain a more representative sample.

Third, the reliance on self-report measures introduces potential susceptibility to common method bias and social desirability bias, despite steps taken to mitigate this (e.g., validated scales, Harman’s test, marker variable). Incorporating multi-informant reports or objective physiological/behavioral measures could strengthen future research. Fourth, while online data collection is efficient, ensuring participant attention and minimizing rushed responses remains a challenge, even with attention checks and completion time monitoring. More robust methods for ensuring data quality in online surveys could be employed. Finally, the study’s focus on Chinese college students limits the direct generalizability of findings to other cultural or demographic contexts, as the expression and impact of these psychological constructs may vary culturally [58].

Building on the present study and its limitations, several avenues for future research emerge. First, longitudinal studies are needed to track the development of these variables over time and examine causal relationships and potential reciprocal effects. Second, experimental intervention studies could directly test whether programs designed to enhance gratitude, positive reappraisal, and peace of mind lead to significant improvements in psychological well-being among students. Third, research should investigate potential moderators of the observed relationships, such as personality traits (e.g., neuroticism, optimism), levels of social support, or specific types of stressors encountered by students, which could inform the tailoring of interventions. Fourth, future studies could benefit from multi-method approaches to measurement, combining self-reports with peer reports, implicit measures, or physiological indicators (e.g., heart rate variability) to provide a more comprehensive assessment, particularly for peace of mind. Finally, cross-cultural research is essential to examine whether the mediating role of peace of mind and the overall model hold in diverse cultural settings, contributing to a more universal understanding of these positive psychological processes.

## Conclusion

In summary, this study provides empirical support for a model linking gratitude and positive reappraisal to psychological well-being among Chinese college students, importantly identifying peace of mind as a significant mediating pathway. Both gratitude and positive reappraisal are associated with higher well-being, and they also appear to foster well-being indirectly by cultivating a sense of inner tranquility and emotional stability. These findings contribute to positive psychology theory by elucidating mechanisms of well-being and offer valuable insights for developing interventions aimed at supporting the mental health of college students navigating demanding educational environments.

## Supplementary Information

Below is the link to the electronic supplementary material.

Supplementary Material 1

## Data Availability

The raw, anonymized dataset generated and analyzed for this study is included as a supplementary information file with this article.

## Abbreviations

AVE: Average Variance Extracted
BC-CI: Bootstrap Confidence Interval
CERQ: Cognitive Emotion Regulation Questionnaire
CFA: Confirmatory Factor Analysis
CFI: Comparative Fit Index
CMB: Common Method Bias
EFA: Exploratory Factor Analysis
EI: Emotional Intelligence
FIML: Full Information Maximum Likelihood
GFI: Goodness-of-Fit Index
GQ-6: Gratitude Questionnaire–6 item
MCAR: Missing Completely at Random
PoMS: Peace of Mind Scale
PWB: Psychological Well-being
RMSEA: Root Mean Square Error of Approximation
SD: Standard Deviation
SEM: Structural Equation Modeling
SPWB: Scale of Psychological Well-being
SRMR: Standardized Root Mean Square Residual
TLI: Tucker-Lewis Index
